# Effect of acetic acid on ethanol production by *Zymomonas mobilis* mutant strains through continuous adaptation

**DOI:** 10.1186/s12896-017-0385-y

**Published:** 2017-08-01

**Authors:** Yu-Fan Liu, Chia-Wen Hsieh, Yao-Sheng Chang, Being-Sun Wung

**Affiliations:** 1 Division of Allergy, Department of Pediatrics, Chung-Shan Medical University Hospital, Taichung, Taiwan; 2 Department of Biomedical Sciences, College of Medicine Sciences and Technology, Chung Shan Medical University, Taichung, Taiwan; 3 Department of Microbiology, Immunology and Biopharmaceuticals, National Chiayi University, Chiayi, Taiwan

**Keywords:** Lignocellulosic hydrolysates, Acetic acid, Bioethanol, *Zymomonas mobilis*, Acid adaptation

## Abstract

**Background:**

Acetic acid is a predominant by-product of lignocellulosic biofuel process, which inhibits microbial biocatalysts. Development of bacterial strains that are tolerant to acetic acid is challenging due to poor understanding of the underlying molecular mechanisms.

**Results:**

In this study, we generated and characterized two acetic acid-tolerant strains of *Zymomonas mobilis* using N-methyl-N′-nitro-N-nitrosoguanidine (NTG)-acetate adaptive breeding. Two mutants, ZMA-142 and ZMA-167, were obtained, showing a significant growth rate at a concentration of 244 mM sodium acetate, while the growth of *Z. mobilis* ATCC 31823 were completely inhibited in presence of 195 mM sodium acetate. Our data showed that acetate-tolerance of ZMA-167 was attributed to a co-transcription of *nha*A from ZMO0117, whereas the co-transcription was absent in ATCC 31823 and ZMA-142. Moreover, ZMA-142 and ZMA-167 exhibited a converstion rate (practical ethanol yield to theorical ethanol yield) of 90.16% and 86% at 195 mM acetate-pH 5 stress condition, respectively. We showed that acid adaptation of ZMA-142 and ZMA-167 to 146 mM acetate increased ZMA-142 and ZMA-167 resulted in an increase in ethanol yield by 32.21% and 21.16% under 195 mM acetate-pH 5 stress condition, respectively.

**Conclusion:**

The results indicate the acetate-adaptive seed culture of acetate-tolerant strains, ZMA-142 and ZMA-167, could enhance the ethanol production during fermentation.

## Background

Lignocellulosic biomass is attractive as a feedstock because of its renewability and remarkable availability in industrial bioconversion. Use of lignocellulosic biomass for ethanol genesis through biochemical processes presents technical difficulties [1]. The main challenge is suggested to be pretreatment and enzymatic hydrolysis steps with which fermentable sugars are released from the biomass. These steps have been suggested to generate a variety of toxic compounds and form a stressful environment which inhibits microbial fermentations [2–4]. Inhibitors include furans, phenols, organic acids, aldehydes and alcohols [5, 6] of these inhibitors, acetic acid is known at the predominant inhibitor formed from lignocellulosic hydrolysates during pretreatment [7]. Toxicity of acetic acid to ethanologens is suggested to be pH based growth inhibition and disruption of pH and anion pool of cytoplasm. Strategies have been developed to overcome such issues for high ethanol production including removal of fermentation inhibitors and use of inhibitor-tolerant strains. However, there is currently no ideal bacterial ethanologen available for use in an industrial setting.

*Zymomonas mobilis*, a Gram-negative facultative bacterium, is considered as an ideal organism for large scale ethanol production. It has several favorable industrial characteristics including high ethanol production, excellent ethanol tolerance, use of the Entner-Doudoroff pathway and a broad range of working pH. Wild type *Z. mobilis* strains are known for a lack of pentose metabolism pathway and sensitivity to inhibitors formed during pretreatment [7–9]. Several mutant strains of *Z. mobilis* have been engineered to overcome substrate limitation and growth inhibition including ZM4/AcR [10], AX101 [11] and ZM4(pZB5) [12]. Techniques employed to obtain mutant *Z. mobilis* including chemical mutagenesis, genetic recombination, metabolic engineering and evolutionary adaptation [13–15]. It is of interest to develop an appropriate approach that facilitate industrial strain improvement.

In this study, two sodium acetate-tolerant mutant of *Z. mobilis* ZMA-142 and ZMA-167 were generated by chemical mutagenesis and adaptive evolution from parental strain *Z. mobilis* ATCC 31823 (ZM481). Full characterization of the cell growth behavior, ethanol fermentation characteristics, and metabolic profiles were conducted.

## Methods

### Bacterial stains and culture conditions

*Zymomonas mobilis* ZM481 (ATCC31823) was chosen and used as a starting strain for adaption. ZM481 was maintained in Rich medium (RM; 20.0 g/L glucose, 10.0 g/L yeast Extract, 2.0 g/L KH_2_PO_4_, pH 5.0) at 30 °C without shaking. Adapted strains were cultured with RM medium containing 100 g/L glucose for investigation on its profiling of cell growth, glucose utilization, and ethanol yield under normal or stress conditions. Bacterial strains were grown overnight, mixed with equal volume of 60%(*w*/*v*) glycerol solution and stored at −80 °C.

### Fermentations

Bacterial strains were revived from glycerol stock in RM broth for 6-8 h, followed by streaking the culture on RM agar plate. Colonies taken from plates were grown for 18 h without shaking at 30 °C in RM broth. Resulting pre-seed cultures were inoculated at a ratio of 2% (*v*/v) into seed medium (100 g/L glucose, 5 g/L yeast extract (Oxoid), 1 g/L MgSO_4_.7H_2_O, 1 g/L (NH_4_)_2_SO_4_and 2 g/L KH_2_PO_4_). Seed cultures were grown at 30 °C without shaking to mid growth phase and subsequently inoculated at 10%(*v*/v) into fermentation medium (FM), which was identical in composition to the seed medium except that final concentrations of up to 196 mM sodium acetate (Ajax Chemicals, NSW, Australia, AR grade). Batch fermentation was conducted in a 5-L jar fermentor (New Brunswick Scientific, Enfield, CT, USA) with 3 L working volume fermenters at controlled pH 5 and temperature 30 °C. Fermentation medium was inoculated (10% *v*/v) with seed culture and incubated with agitation at 200 rpm. The culture medium was FM contained with/out 195 mM sodium acetate and the pH in 5.0. The initial number of cells (CFU) for all tests was approximately equal 2.0 × 10^7^ mL-1.

### N-methyl-N-nitro-N-nitrosoguanidine (NTG) treatment

The acetate- tolerant mutant of *Z. mobilis* was selected and mutated with NTG. 1 mL of NTG solutions (0.4 mg/mL) were added to 9 mL of the cell suspension of ZM481. The mixture was incubated at 37 °C and agitated at 200 rpm for 30 and 45 min, followed by a centrifugation at 8000×g for 10 min. The pellet was washed thrice with 3 mL of 0.85% NaCl and then resuspended in 1 mL of the seed medium. The culture was incubated for 24 h at 30 °C and subsequently repetitively cultivated in the adaptive evolution medium.

### Adaptive evolution experiments

The concentrations of acetic acid in lignocellulosic hydrolysates are known in the range from 16.7 to 258 mM (1, 23). In this study, initial concentration of acetate for adaption was 195 mM and the concentrations increased up to 270 mM at pH 5. The procedures of adaptive mutation were descripted in brief as follows. Firstly, NTG treated ZM481 was grown in RM broth in the absence of sodium acetate acetic acid at pH 7.0 and 30 °C without shaking overnight. The culture was inoculated into a new 10-ml Falcon tubes containing 5 ml of RM broth supplemented with 195 mM sodium acetate, followed by transferring into the same medium using the method of serial batch transfer for 3 times. The resulting culture was plated onto RM agar plate supplemented with 195 mM sodium acetate and incubated at 30 °C. 400 individual colonies were selected and subject to assessment for cell growth under stress conditions as 220, 250 and 270 mM sodium acetate pH 5, respectively. Two strains, namely ZMA-142 and ZMA-167 were obtained and selected for further analyses.

### Analytical methods

The concentration of glucose was determined using YSI 2300 STAT plus analyser (Yellow Springs Instrument Co., USA). Ethanol concentration was estimated using gas chromatography with a glass 4 mm ID 32 m Porapak Q 100/120 mesh column operated isothermally with N_2_ as carrier and a flame ionisation detector. Peak areas were determined with an integrator.

### RNA preparation and analysis

Total RNA was prepared as previously described (24). Briefly, cells were harvested by centrifugation, followed by a total RNA extraction using TRIzol reagent (Invitrogen). Purification of total RNA was perfoemd using NucleoSpin® RNA clean-up (MACHEREY-NAGEL, Germany) according to the manufacturer’s instructions. The RNA quality and quantity were assessed by formaldehyde agarose gel electrophoresis and measured by calculating absorbance ratio at both 260/280.

### Reverse transcription PCR

Reverse transcription was carried out using four primers, ZMO0117F (TGTGATGGTATC AAAAGCGGTC), ZMO0117R (CCAAATCGGTGACACGGAA), ZMO0119F (CTGCTCTTATCCGCCCTTC), ZMO0119R (GGAAAGAAGCCAGATGTCCC) in combinations. Reverse transcription PCR was performed using Eppendorf® Mastercycler Personal within Super 2 RT-PCR Mix (Protech). The reverse transcription PCR conditions were set as following: 30 min at 45 °C, 10 min at 95 °C, 25 cycles at 95 °C for 30 s, followed by 55 °C for 30 s and 72 °C for 70 s, and final extension at 72 °C for 10 min. The reverse transcription PCR product was subject to electrophoresis in 1.5% agarose gel incorporating with Omics 100 bp Plus DNA RTU Ladder.

### Genomic DNA capture and sequencing

Genomic DNA samples measured with O.D. 260/280 in the range 1.8 ~ 2.0, and quantity ratio by Qubit versus NanoDrop over 0.7 were considered as acceptable for further processing. The sequencing library construction and sequencing were performed by Welgene Biothech Co., Ltd., Taiwan. Isolated DNA was sonicated by Misonix 3000 sonicator to the size ranging from 400 to 500 bp. DNA sizing was checked by bioanalyzer DNA 1000 chip (Agilent TechnologiesTM). All subsequent steps were performed on the instrument, including end-repaired, A-tailed and adaptor-ligated. After library construction, samples were mixed with MiSeq Reagent Kit (600-cycle) and loaded onto Illumina Solexa Miseq platform, which was performed by Welgene Biotech Co., Ltd. (Taipei, Taiwan) [16]. Automated cluster generation and a 2 × 300 bp paired-end sequencing run were performed.

### Sequence analysis and alignment

The Miseq reads were trimmed and filtered by Illumina protocols to ensure the high quality data for all following analyses. To evaluate the raw reads quality by CLC-relative standard NGS packages was used to trim reads that did not achieve sufficient quality PHRED score > 20), excluding short sequences (<30 bp). Sequences were aligned to the ZM4 ATCC31821 (accession number, NC006526) reference genome using BWA-SW read alignment programs [17, 18]. To visualize reads coverage and insertion/deletion junctions, estimate transcripts structure and evaluate different expression patterns in the different mutants by Integrative genomics viewer (IGV) [19].

## Results

### Improvement of acetate tolerance through NTG mutagenesis

ZM481 is known for its limited growth in presence of high concentration of acetate. To obtain a library of mutants of ZM 481, chemical mutagenesis using NTG was performed. Stepwise adaptive evolution was employed for screening acetate-tolerant mutants. NTG-treated ZM481 was repetitively cultured in the adaptive evolution medium with stepwise increasing concentrations of sodium acetate up to 270 mM. In total, nine rounds of mutagenesis and selection were performed for the improvement of acetate tolerance. A total of 400 mutants were obtained from NTG-treated ZM 481 grown in present of 195 mM sodium acetate. Adapting ZM481 to serial concentrations of sodium acetate, there were 20 mutants identified with abilities to grow in a condition of 270 mM sodium acetate. Of the 20 mutants, two mutants, namely ZMA-142 and ZMA-167, were genetically stable for over 20 generations and therefore chosen for further characterization.

### Characterization of acetate-tolerant ZMA 142 and ZMA 167

**Fig. 1:**
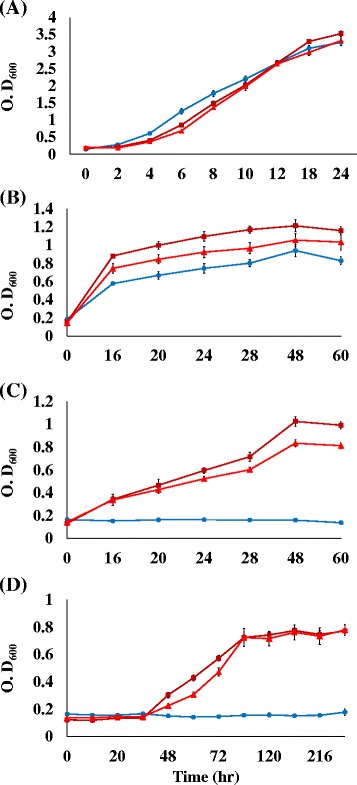
Comparison of growth by the acetate-tolerant mutant ZMA-167(■), ZMA-142(▼) and ZM481(●) with 0 mM (**a**), 146 mM (**b**), and 195 mM (**c**) and 244 mM (**d**) sodium acetate (500-mL flask, pH 5.0, 30oC). The data were presented in mean values of triplicate experiments

We next characterized the growth features of ZM481 and two acetate-tolerant mutants, ZMA142 and ZMA-167, in culture supplemented with sodium acetate at serial concentrations of 0, 146, 195 and 243 mM. As shown in Fig. 1A, the differences in cell growth among three strains after 12-h under normal FM culture condition (pH 5.0) were insignificant, showing that ZM481, ZMA-142 and ZMA-167 reached maximum optical densities at OD600 of 3.5, 3.3 and 3.5, respectively. The data showed that increasing concentrations of sodium acetate led to a reduction in cell growth rate in three strains. ZMA-167 exhibited higher growth rate at concentrations of 146 and 195 mM after an incubation of 60 h compared with those of ZMA-142 (Fig. 1b and c). Interestingly, the growth rates of two mutants at high concentration of acetate (243 mM) were identical after 240 h incubation, whereas ZMA-167 exhibited better growth than ZMA-142 in first 60 h incubation. The growth of ZM481 was completely inhibited by sodium acetate at a concentration of 195 mM.

### Batch fermentations with ZM481 and selected acetate-tolerant mutants

**Fig. 2:**
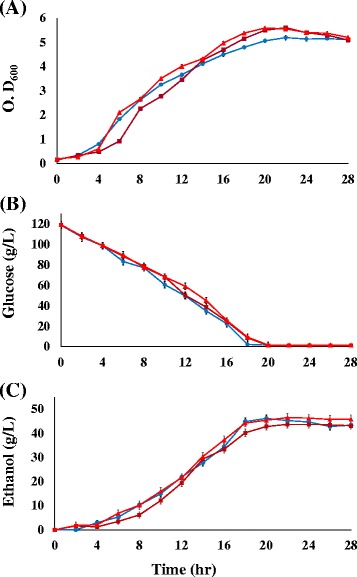
Profiling of cell growth (**a**), glucose utilization (**b**), and ethanol yield (**c**) of the acetate-tolerant mutant ZMA-167(■), ZMA-142(▲) and ZM481(●) grown in 5 L fermenter(pH 5.0, 30oC) glucose utilization (**b**), and ethanol yield (**c**) of the acetate-tolerantwere presented in mean values of triplicate experiments

To evaluate the performances of the ZM481 and the selected acetate-tolerant mutants in ethanol production, batch fermentations with these strains were performed. Sodium acetate concentrations were chosen according to the highest concentrations tolerated by ZM481 and two selected mutants. Glucose consumption and ethanol production were measured using HPLC. As shown in Fig. 2, in normal culture conditions, the fermentative performances of ZM481 and two mutants were comparable as in sugar utilization rate and ethanol yield rate. Ethanol concentration of ZM481, ZMA-142 and ZMA-167 were 46.1, 46.5, and 43.7 g ethanol/L, respectively. The ratio of ethanol produced to moles of glucose consumed in ZM481, ZMA-142 and ZMA167 was 0.43, 0.47 and 0.44, respectively. The results revealed that ZMA-142 or ZMA-167, in 5-L batch fermentation with sodium acetate-adaptive seed culture, exhibited an accelerated fermentation process compared with that of normal seed culture (Table 1).

**Table 1:** Effect of acetate-adaptive seed culture on ZMA-142 and ZMA-167 in a 5 L fermenter using medium containing 120 g glucose/L, 195 mM sodium acetate and controlled at pH 5.0

Strains	Fermentation time (hr)	Glucose utilization (g/L)	Ethanol Production (g/L)	*q*_*s*_max^a^	*q*_*P*_max^b^	*Yp/s* ^c^	Conversion Rate (%)^d^
Seed culture with RM; Fermentation in FM							
ZM 481	20	107.2	46.1	5.4	2.3	0.43	84.0
ZMA-142	22	99.2	46.5	4.5	2.1	0.47	91.8
ZMA-167	22	100.0	43.7	3.9	1.7	0.44	86.0
Seed culture with RM; Fermentation in FM, 195 mM NaOAc, pH 5							
ZM 481	64	11.40	2.3	0.2	0.04	0.20	39.1
ZMA-142	56	103.9	48.0	1.86	0.9	0.46	90.2
ZMA-167	64	100.7	44.3	1.57	0.7	0.44	86
Seed culture with RM, 146 mM NaOAc; Fermentation in FM, 195 mM NaOAc, pH 5							
ZM 481	48	12.4	2.9	0.3	0.1	0.23	45.0
ZMA-142	36	97.2	60.8	2.7	1.7	0.63	122.4
ZMA-167	48	86.6	47.4	1.8	1.0	0.55	107.2

^a^qs max: Total sugar utilization rate (g l-1 h-1)

^b^qp max: Ethanol production rate (g l-1 h-1)

^c^Yp/s: Ethanol yield on total sugars (g g-1)

^d^Conversion Rate(%) = (Yp/s) x 100%/(Theorical Yp/s), Theorical Yp/s = (46*2)/180.16 = 0.51143

**Fig. 3:**
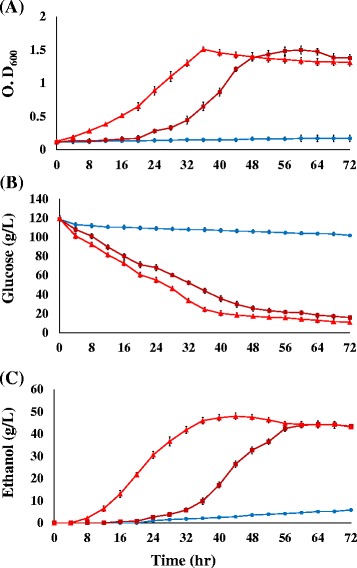
Profiling of cell growth (**a**), glucose consumption (**b**), and ethanol yield (**c**) of the acetate-tolerant mutant ZMA-167(■), ZMA-142(▲) and ZM481(●) grown in 5 L fermenter(pH 5.0, 30 °C, 200 rpm) with 120 g/L glucose under 195 mM sodium acetate stress condition. The data come from mean values of triplicate experiments

We next examined fermentative pattern of ZM481 and two selected mutants in response to high acetate concentrations of 195 mM in a 5-L fermenter with 120 g glucose/L. The results revealed that ZMA-142 exhibited higher growth rate than that of ZMA-167, whereas the growth of ZM481 was significantly inhibited (Fig. 3a). In addition, the glucose consumption rates of ZMA-142 and ZMA-167 were declined to constant levels after 44 and 56 h incubation, respectively (Fig. 3b). The data showed that ZMA-142 reached the highest ethanol concentration (48 g/L) in 44 h, whereas highest ethanol production was observed in mutant ZMA-167 after an incubation of 64 h.

**Fig. 4:**
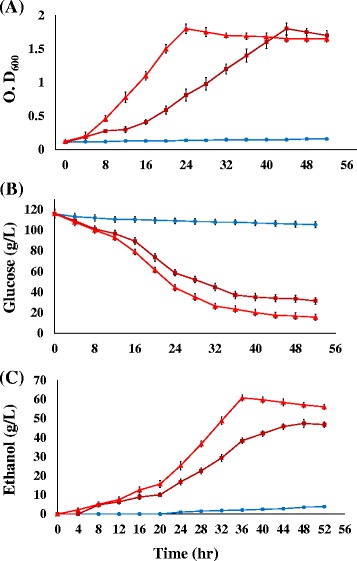
Profiling of cell growth (**a**), glucose utilization (**b**), and ethanol yield (**c**) of the acetate-tolerant mutant ZMA-167(■), ZMA-142(▲) and ZM481(●) grown in 5 L fermenter(pH 5.0, 30 °C, 200 rpm) 120 g/L glucose under 195 mM sodium acetate stress condition. Inoculation with 146 mM sodium acetate-adaptive seed culture. The data come from mean values of triplicate experiments

We determined the effects of seed culture on ethanol production of ZMA-142 and ZMA-167 in response to high sodium acetate concentration. Seed culture adapted to 146 mM sodium acetate with an inoculum size of 10% (*v*/v) was added to a medium containing 195 mM sodium acetate. We found that ZMA-142 and ZMA-167 reached the stationary phase of cell growth in 24 and 44 h, respectively (Fig. 4a). The data showed that glucose consumption of ZMA-142 was 96.5 g/L and maximum ethanol concentration was 60.8 g/L in 36 h, whereas ZMA-167 reached its maximum glucose consumption in 48 h and produced ethanol of 47.4 g/L (Fig. 4b and c). In addition, ethanol yield and ethanol conversion rate of ZMA-142 and ZMA-167 after 36 h and 48 h fermentation were 0.6 g/g and 122.4%, 0.55 g/g and 107.2%, respectively (Table 1). Our data showed that ZMA-142 and ZMA-167 exhibited relatively higher the glucose uptake rate and ethanol fermentation performance in acetate-adaptive seed culture used fermentation.

### Genomic variants for improvement of acetate tolerance

**Fig. 5:**
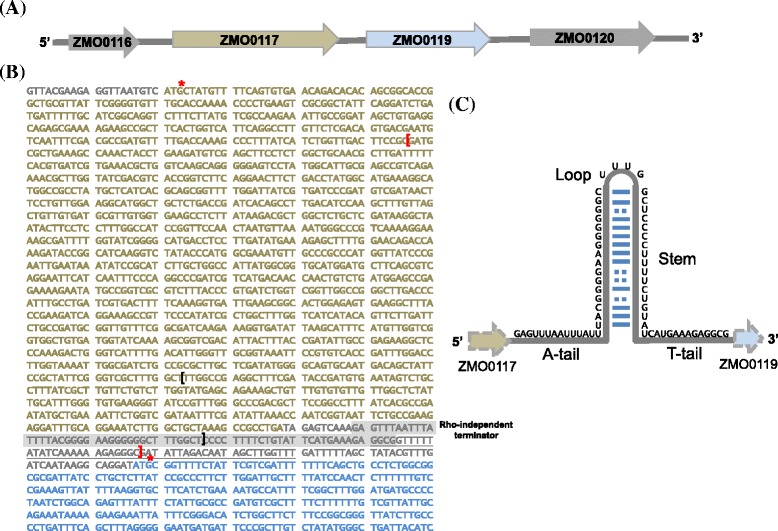
The Rho-independent terminator between the ZMO0117 and ZMI0119 genes in the Z. mobilis ZM4. **a** The genomic organization of ZMO0117 (ORF is shown in dark grey) and ZMO0119 (ORF is shown in light grey) was presented. **b** The translation start sites of the both genes as identified by genome annotation were marked with asterisks. The conserved Rho-independent terminator was predicted by TransTermHP server and shaded in color grey. The sequence was predicted as the putative promoter region of ZMO0119 using the Neutral Network Promoter Prediction (NNPP) server and underlined in black. The color black of square brackets indicated the regions of DNA deleted in the ZMA-167 in this study comparison with genome of ZM4, ZM481 and ZMA-142. The primer sequences used were double-underlined. **c** The schematic of the Rho-independent terminator motif for which TransTermHP search (HP score is −3.141 and 100% confidence). The terminators conserved sequence consist of a short stem-loop hairpin (standard Watson-Crick pairs and Wobble base pairs G-U shown as the grey and dotted lines, respectively) followed by thymine-rich region on their 3′ side

**Fig. 6:**
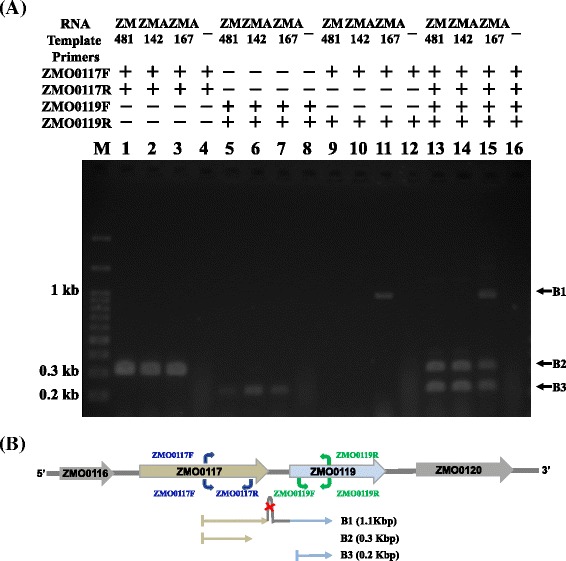
RT-PCR detection of ZMO0117 and ZMO0119 mRNA from the acetate-tolerant mutant ZMA-142, ZMA-167 and ZM481. **a** Total RNA from the acetate-tolerant mutant ZMA-142(treated with 196 mM sodium acetate)(lane 2, 6, 10 and 14), ZMA-167 (treated with 196 mM sodium acetate)(lane 3, 7, 11 and 15) and ZM481 (without treated with sodium acetate) (lane 1, 5, 9 and 13) cells in the middle exponential phase were RT-PCR performed with various combinations of primers (ZMO0117F, ZMO0117R, ZMO0119F and ZMO0119R, respectively), in three independent reactions. Ten micrograms of each RNA was electrophoresed through 1% agarose–2.2 M formaldehyde gels and stained with ethidium bromide. The negative (lane 4, 8, 12, 16) controls sterile water. Lane M contained 0.1 ~ 3 kb markers. **b** Schematic representation of the ZM481, ZMA-142 and ZMA-167 chromosome containing the ZMO0117 and ZMI0119 genes. Arrows indicate positions of the predicted transcripts, B1 (transcipted from ZMO0117 to ZMO0119), B2 (ZMO0117), and B3(ZMO0119)

To determine the genomic variants in acetate-tolerant mutants, next-generation sequence (NGS) by reversible dye terminators sequencing, Illumina solexa Miseq, was employed following combine with genome-wide bioinformatics approaches. Based on the all genomic DNA libraries were aligned on the same strand as the targeted feature genes of reference genome using SAMtools package v.1.19, ZMA-167 had a deletion of 303-bp, which was confirmed using PCR. The deletion truncated 3′ part of ZMO0117 by 255-bp and upstream of ZMO0119 (Fig. 5). TransTerm HP search showed that there was a putative ρ-dependent terminator 66 bp downstream of ZMO0117 in ZM481 (HP score is −3.141 and 100% confidence), whereas ZMA-167 had truncated ρ-dependent terminator by 28 bp. Using the Neutral Network Promoter Prediction (NNPP), server to predict the sequence of putative promoter region of ZMO0119 in ZMA-167. The putative promoter region of ATCC 31823 ZMO0117 was 60-bp, which was the same with those in ZMA-167. We next analyzed the 0.3-Kbp deletion in ZMA-167 using reverse-transcription PCR. Using the PCR primer set, ZMO0117F and ZMO0117R, a 0.3-Kbp RT-PCR product from ZMA-167 and ZM481 were obtained respectively (Fig. 6a, lane 1, 2, 10 and 11). Use of primer set, ZMO0119F and ZMO0119R, resulted in 0.2-Kbp RT-PCR products from ZMA-167 and ZM481 respectively (Fig. 6a, lane 4, 5, 10 and 11). Interestingly, a 1.1-Kbp RT-PCR product was obtained from ZMA-167 with a primer set of ZMO0117F and ZMO0119R, whereas there was no product generated from ZM481 (Fig. 6a, lane 7, 8, 10 and 11). Our RT-PCR data showed that a ZMO0117-ZMO0119 co-transcription product of the 0.3-Kbp deletion ZMA-167 was detected 195 mM sodium acetate culture condition (Fig. 6). In addition to 0.3-Kbp deletion in ZMA-167, there was no genome change in this region in ZMA-142 compared with ZM481 parental strain.

## Discussion

In the present study, we reported the inhibitory effects of acetate and low pH on an ethanol-tolerant strain. Using combination of NTG mutagenesis and adaptive evolution, we obtained two acetate-tolerant mutants exhibiting favorable ethanol production under high-acetate condition. We found that the acetate-tolerant property of mutant ZMA-167 was attributed to a deletion of sequence of terminator of *nha*A encoding hydroxylamine reductase.

*Z. mobilis* is known for its features of rapid glucose uptake and favorable alcohol production. Several *Z. mobilis* strains have been developed to have high ethanol tolerance and to use a wide range of sugars [9, 15, 20–22]. With improved strains of *Z. mobilis*, the main challenges have become generating an ethanologen tolerant to inhibitors formed and released during the pretreatment and hydrolysis process. Many inhibitors have been reported including furans, phenols, organic acids and alcohols [7, 23–25]. Acetic acid has been shown to be a ubiquitous and strong inhibitor on ethanologen growth during ethanol fermentation [26–28]. Our results that the growth of ZM481 was decreased corresponding to increasing concentration of acetate were in consistent with previous studies. pH value has been shown to have an impact on inhibitory effect of acetic acid on the growth of ethanologen [29–31]. In the present experimental setting, the percent dissociation of acetic acid in the growth medium at pH 5 was 64%, resulting in 36% uncharged, undisscoated acetic acid that across cell membrane freely and compromise the biological balance. In addition to acetic acid, sodium ion has been suggested to synergistically contribute to decreased cell growth of *Z. mobilis* [7, 31]. Many genetic approaches and strategies have been employed to improve tolerance to variety of inhibitors in *Z. mobilis* [32]. Rogers et al. reported an acetate-tolerant mutant of *Z. mobilis* (AcR) generated using chemical mutagenesis and adaptation [10]. Several strains of *Z. mobilis* have been generated for tolerance to inhibitors by transposon mutagenesis [33–36]. Evolutionary adaptation has been employed to improve fermentation capability and resistance to inhibitory stress [29]. In this study, we obtained two mutants, ZMA-142 and ZMA-167 generated from ZM481, which exhibited improved acetate tolerance with favorable ethanol production at low pH. Our results suggest that chemical mutagenesis incorporating with adaptive evolution represents a practical technique to develop strains adapted to various inhibitory substrates and stress. However, molecular responses of two mutants to acetic acid and glucose are dynamic and complicated, which require further studies to elucidate.

Acetic acid toxicity is suggested to hinder the ethanologenesis by *Z. mobilis*. Acetate-resistant mutant (AcR) of *Z. mobilis* has been generated from ZM4, which has high ethanol production in the presence of 20 g/L sodium acetate [10]. The genome of ZM4 has been sequenced and its annotation has been improved [17, 37]. A recent study has identified the mutant loci that contribute to sodium acetate tolerance in the AcR strain [31]. Yang et al. has reported that a regulator Hfq protein acting as an RNA chaperone was involved in the acetate-tolerance in *Z. mobilis* [38]. In the present study, two mutants generated were examined for genome changes in comparison with their parental strain ZM481. We identified a 303-bp of deletion within the ρ-dependent terminator putative sequences of ZMO0117 and upstream of ZMO0119 in ZMA-167 mutant. AcR mutant has reported to have a 1.5 kbp of deletion in ZMO0117- ZMO0119 resulting in acetate-tolerant phenotype [31]. Our results showing a deletion in terminator sequence of ZMO0117 and upstream of ZMO0119 in ZMA-167 are in agreement with previous study. It is suggested that elimination of a potential ZMO0117 transcriptional terminator lead to an over-expression of ZMO0119 encoding Na+/H+ antiporter by promotor of ZMO0117 in ZMA-167. In addition to ZMO0119, deletion of 303 bp in ZMA-167 resulted in removal of terminator putative sequences of ZMO0117 encoding hydroxylamine reductase. Hydroxylamine reductase is suggested to function as scavenger of other intermediates of nitrate ammonification in bacteria [39, 40]. It is implied that removal of terminator of ZMO0117 putatively results in high expression of hydroxylamine reductase, leading to a resistance to stress. However, further studies are required to elucidate the role of hydroxylamine reductase in acetate tolerance.

## Conclusion

In conclusion, a combination of NTG mutagenesis and adaptive evolution represents an ideal strategy to generate mutants in response to environmental stress such as acetic acid. Taken the approach, two acetate-tolerant mutant *Z. mobilis* ZMA-142 and ZMA-167 exhibited enhanced tolerance to acetate and significantly improved fermentation performances. The mutants are suggested to have potential for industrial bioconversion process.

## Abbreviations

AcR: Acetate-resistant mutant
CGS: Comparative Genome Sequencing
FM: Fermentation medium
HPLC: High performance liquid chromatography
Kbp: Kilo base pairs
NNPP: Neutral Network Promoter Prediction
NTG: N-methyl-N′-nitro-N-nitrosoguanidine
OD: Optical density
PCR: Polymerase chain reaction
RM: Rich medium
RT-PCR: Reverse transcription PCR
